# PON1 and Neurodevelopment in Children from the CHAMACOS Study Exposed to Organophosphate Pesticides *in Utero*

**DOI:** 10.1289/ehp.1002234

**Published:** 2010-08-19

**Authors:** Brenda Eskenazi, Karen Huen, Amy Marks, Kim G. Harley, Asa Bradman, Dana Boyd Barr, Nina Holland

**Affiliations:** 1 Center for Environmental Research and Children’s Health, School of Public Health, University of California, Berkeley, Berkeley, California, USA;; 2 Emory University, Rollins School of Public Health, Atlanta, Georgia, USA

**Keywords:** biomarkers, Child Behavior Checklist, children, DAPs, farmworker, genetic susceptibility, *in utero* exposure, mental development, Mexican Americans, neurodevelopment, organophosphates, paraoxonase, pervasive developmental disorder, pesticides, PON1

## Abstract

**Background:**

Paraoxonase 1 (PON1) detoxifies oxon derivatives of some organophosphate (OP) pesticides, and its genetic polymorphisms influence enzyme activity and quantity. We previously reported that maternal urinary concentrations of dialkyl phosphate (DAP) metabolites, a marker of OP pesticide exposure, were related to poorer mental development and maternally reported symptoms consistent with pervasive developmental disorder (PDD) in 2-year-olds participating in the Center for the Health Assessment of Mothers and Children of Salinas (CHAMACOS) study.

**Objective:**

We determined whether PON1 genotypes and enzyme measurements were associated with child neurobehavioral development and whether PON1 modified the association of *in utero* exposure to OPs (as assessed by maternal DAPs) and neurobehavior.

**Methods:**

We measured DAP concentrations in maternal urine during pregnancy, *PON1_192_* and *PON1_−108_* genotypes in mothers and children, and arylesterase (ARYase) and paraoxonase (POase) in maternal, cord, and 2-year-olds’ blood. We assessed 353 2-year-olds on the Mental Development Index (MDI) and Psychomotor Development Index (PDI) of the Bayley Scales of Infant Development and queried their mothers on the Child Behavior Checklist to obtain a score for PDD.

**Results:**

Children with the *PON1_−108T_* allele had poorer MDI scores and somewhat poorer PDI scores. Children were less likely to display PDD when they or their mothers had higher ARYase activity and when their mothers had higher POase activity. The association between DAPs and MDI scores was strongest in children with *PON1_−108T_* allele, but this and other interactions between DAPs and PON1 polymorphisms or enzymes were not significant.

**Conclusion:**

PON1 was associated with child neurobehavioral development, but additional research is needed to confirm whether it modifies the relation with *in utero* OP exposure.

The paraoxonase 1 (PON1) enzyme detoxifies organophosphate (OP) pesticides, which are known neurotoxicants at high doses (Costa et al. 2005). PON1 also inhibits low-density lipoprotein oxidation, a marker of oxidative stress (Li et al. 2003). Several common polymorphisms in the coding (e.g., *PON1_192_*) and promoter (e.g., *PON1_−108_*) regions of the *PON1* gene influence both the quantity and catalytic efficiency of the PON1 enzyme (Brophy et al. 2001; Li et al. 2000), measured against specific substrates, such as arylesterase (ARYase) and paraoxonase (POase) activity.

PON1 polymorphisms and/or enzyme measurements have been associated with various diseases of the nervous system, including Alzheimer’s disease (Erlich et al. 2006; Leduc and Poirier 2008; Paragh et al. 2002), brain tumors (Kafadar et al. 2006), vascular dementia (Paragh et al. 2002), amyotrophic lateral sclerosis (Saeed et al. 2006; Slowik et al. 2006), ischemic stroke (Voetsch et al. 2002, 2004), and Parkinson disease (Zintzaras and Hadjigeorgiou 2004). Gulf War syndrome, which some have hypothesized may be attributable to exposure to an OP agent (Golomb 2008), has also been associated with low PON1 ARYase (Haley et al. 1999) and POase (Mackness et al. 2000) measurements. PON1 polymorphisms or enzyme activity may also play a role in psychiatric disease such as schizophrenia (Kucukali et al. 2008), depression (Lawlor et al. 2007), and anxiety (Sklan et al. 2004). In addition, *PON1_192_* genotype and PON1 enzyme measurements have been associated with childhood autism (Pasca et al. 2006, 2010), at least in certain populations (D’Amelio et al. 2005).

An individual’s susceptibility to the effects of specific OP pesticide exposure may be determined by their *PON1* genotypes and expression (Li et al. 2000). PON1 enzyme measurements vary widely in humans (Cole et al. 2003; Costa et al. 2005; Holland et al. 2006), and measurements in fetuses and children up to at least 7 years of age are much lower than those in adults (Chen et al. 2003; Furlong et al. 2006; Huen et al. 2010), thus presenting a potential period of greater vulnerability to OP pesticide toxicity and oxidative stress. Transgenic newborn mice expressing *PON1_192Q_* showed greater inhibition of brain acetylcholinesterase after chlorpyrifos oxon exposure than did those with *PON1_192R_* (Cole et al. 2003). In a birth cohort study from New York, an association between abnormal neonatal reflexes and maternal dimethyl OP pesticide exposure (as measured by urinary metabolites) was found only in children with lower levels of POase expression, although an association between abnormal reflexes and urinary diethyl phosphate (DE) metabolites was observed regardless of POase expression (Engel et al. 2007).

In our longitudinal birth cohort, the Center for Health Assessment of Mothers and Children of Salinas (CHAMACOS) study, we previously reported high exposure to OP pesticides, as measured by urinary dialkyl phosphate (DAP) metabolite levels, among pregnant women living in the agricultural Salinas Valley of California, relative to levels in women of reproductive age (18–40 years) participating in the National Health and Nutrition Examination Survey (NHANES) (Bradman et al. 2005). We also observed associations between maternal DAP levels during pregnancy, particularly dimethyl phosphates (DMs; resulting from exposure to DM pesticides), and CHAMACOS 2-year-olds’ mental development as assessed on the Bayley Scales of Infant Development (Bayley 1993). Maternal report of symptoms of pervasive development disorder (PDD) in the clinical range on the Child Behavior Checklist (CBCL) were also associated with maternal DAP levels (Eskenazi et al. 2007).

In the present study, we estimated associations between neurodevelopmental outcomes and *PON1* genotypes and with PON1 enzyme activity measured in children at birth and at 2 years of age and in mothers at delivery. We also examined whether PON1 modified the associations we previously observed between the mothers’ DAP concentrations during pregnancy and their children’s neurobehavioral development at 2 years of age (Eskenazi et al. 2007).

## Materials and Methods

### Study subjects

The CHAMACOS study is a longitudinal birth cohort study of the effects of exposures to pesticides and other environmental chemicals on neurodevelopment, growth, and respiratory disease in children from primarily Latino farmworker families in the Salinas Valley, California (Eskenazi et al. 2003). Located in Monterey County, the Salinas Valley is an intensive agricultural area where > 235,000 kg of OP pesticides are applied annually (California Department of Pesticide Regulation 2005). A total of 601 pregnant women were enrolled in the CHAMACOS study, and 528 delivered newborns. Mothers in the CHAMACOS cohort were primarily low-income, Mexican-born, Spanish-speaking women who were farmworkers themselves or lived with farmworkers. In this analysis, we include those mothers who had measured levels of urinary DAP metabolites during pregnancy and whose children were followed up to 2 years of age (*n* = 371). Most women (*n* = 351) and children (*n* = 369) provided a blood specimen that was genotyped for *PON1_192_* and *PON1_−108_*, and a smaller portion of these mothers and children had adequate blood samples for PON1 enzyme measurements (*n* = 304, 266, and 250 for mothers, umbilical cord, and 2-year-olds, respectively) (Huen et al. 2009a, 2010). Study protocols were approved by the University of California, Berkeley, Committee for the Protection of Human Subjects. Written informed consent was obtained from all mothers for themselves and their children.

### Maternal interviews and neurobehavioral assessments

Information on different covariates was collected from maternal interviews and medical record review. Women were interviewed twice during pregnancy (mean = 13.4 and 25.8 weeks’ gestation), shortly after delivery, and when children were 6 months and 1 and 2 years of age. Interviews were conducted in Spanish or English by bilingual, bicultural interviewers. Mothers were administered the Peabody Picture Vocabulary Test (PPVT) (Dunn and Dunn 1981) at the 6-month visit to assess their scholastic/cognitive abilities, and the Center for Epidemiologic Studies Depression Scale (CES-D) (Radloff 1977) at the 1-year visit. The Infant-Toddler Home Observation for Measurement of the Environment (HOME) instrument (Caldwell and Bradley 1984), a measure of the home and social environment, was completed when the children were 6 months and 1 year of age, and 32 of 45 items were completed at 2 years. Prenatal and delivery medical records were abstracted by a registered nurse.

Children were assessed at 2 years of age on the Bayley Scales of Infant Development, 2nd edition (Bayley 1993), a test of developmental functioning of infants and young children comprising two subscales: the Mental Development Index (MDI), which characterizes a variety of cognitive abilities, and the Psychomotor Development Index (PDI), which characterizes large-muscle and fine-motor coordination. Both scales were administered in Spanish and/or English by psychometricians blind to exposure. Psychometricians were trained using standardized protocols and were supervised for quality assurance by a clinical neuropsychologist. Assessments were performed in a private room at the CHAMACOS research office or in a recreational vehicle (RV) modified to be a mobile testing facility. Children were assessed on average (mean ± SD) at 24.6 ± 1.1 months. Each scale is standardized by age to mean ± SD = 100 ± 15.

At the time of the child’s 2-year assessment, the mother was administered the CBCL for ages 1.5 to 5 years, a 99-item tool to assess children’s emotional/behavioral problems and competencies (Achenbach and Rescorla 2000). The CBCL has been widely used in cross-cultural research and collects data on a range of behavior problems, yielding scores for several syndrome scales and five scales designed to be consistent with *Diagnostic and Statistical Manual of Mental Disorders, 4th ed.* (DSM-IV) diagnoses (American Psychiatric Association 2000). For this study, we focus on the results of the DSM-IV–oriented pervasive developmental disorder (PDD) scale, which includes such items as “avoids eye contact,” “rocks head, body,” and “unresponsive to affection,” which are considered consistent with Asperger’s disorder and autistic disorder (Achenbach and Rescorla 2000). A score considered of “clinical” significance is > 97th percentile of the national normative sample.

### OP pesticide exposure: DAP metabolites

Urine specimens were collected from the mother twice during pregnancy. Urine was aliquoted and stored at −80°C until shipment on dry ice to the Centers for Disease Control and Prevention (Atlanta, GA), where specimens were analyzed using gas chromatography/tandem mass spectrometry and quantified using isotope dilution calibration (Bravo et al. 2002) for six DAP metabolites: three DMs (dimethylphosphate, dimethylthiophosphate, dimethyldithiophosphate) and three DEs (diethylphosphate, diethylthiophosphate, and diethyldithiophosphate) (Bradman et al. 2005). These six metabolites represent the by-products of approximately 80% of OP pesticides used in the Salinas Valley. The most commonly used DM pesticides in the Salinas Valley are malathion and oxydemeton-methyl, and the most commonly used DE pesticides are diazinon and chlorpyrifos. Values below the limit of detection (LOD) were assigned a value of 
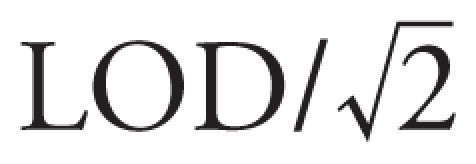
.

### PON1 genotype and enzyme measurements

Maternal blood and fetal umbilical cord blood were collected at the time of delivery, and child blood samples were collected at 2 years of age using BD Vacutainers (Becton, Dickinson and Company, Franklin Lakes, NJ) as previously described (Holland et al. 2006). Heparinized plasma was used to assess PON1 substrate-specific activities at each time point, and maternal and child genotyping was conducted using DNA isolated from blood clots.

The coding polymorphism *PON1_192_* was genotyped using the Taqman real-time polymerase chain reaction (PCR) method as previously described (Holland et al. 2006) using probes custom-designed by Applied Biosystems, Inc. (Foster City, CA). The promoter single-nucleotide polymorphism (SNP) *PON1_−108_* assay required a two-part nested PCR strategy, where the region surrounding the SNP was preamplified using nonallelic flanking primers. The forward primer sequence was 5′-ATAGACAAAGGGATC GATGGGCG-3′, and the reverse primer sequence was 5′-TTTGGCTGAAAGT GCTGAGCTCCTG-3′. The amplicon was then diluted and used as the template for the Amplifluor assay (Flowgen Biosciences Ltd., Nottingham, UK). Quality assurance procedures for genotyping *PON1* SNPs included assessment of randomly distributed blank samples and duplicates with independently isolated DNA from the same subjects. Repeated analysis (4% of samples) in several runs showed a high degree (> 99%) of concordance. All discrepancies were resolved with additional genotyping.

PON1 enzyme activities toward paraoxon (POase) and phenyl acetate (ARYase) were determined using spectrophotometric methods as described by Huen et al. (2009b). The ARYase assay is a quantitative measure of PON1 enzyme level (Connelly et al. 2008; Kujiraoka et al. 2000), which is mostly affected by the promoter polymorphism *PON1_−108_*. In contrast, the POase substrate-specific assay reflects both quantity and catalytic efficiency of the PON1 enzyme and is affected primarily by the coding polymorphism *PON1_192_*. We refer to enzyme quantity, reflected by ARYase, as PON1 enzyme levels and to POase as PON1 enzyme activity. When we refer to both, we use PON1 measurements. All assays were performed in triplicate. Quality assurance included assessment of repeat samples and internal controls. The average coefficient of variation (CV) for repeated samples ranged from 6–9%, and interassay variability, as measured by the average CV for internal control samples, was between 7% and 9% [for more details on validation of these assays for longitudinal studies, see Huen et al. (2009b)].

### Statistical analysis

*PON1* genotypes were examined categorically with two indicator variables per model: For *PON1_−108_*, the CC genotype was the reference, and for *PON1_192_*, the RR genotype was the reference. Supplemental analyses were performed classifying genotypes as ordinal variables (none, one, or two T alleles of *PON1_−108_* or Q alleles of *PON1_192_*). ARYase and POase activity in cord, maternal pregnancy, and blood from 2-year-olds was examined categorically in tertiles and continuously, with values normalized by dividing each value by the SD of the range of values.

DAP metabolite concentrations (nanomoles per liter) were summed and transformed to the log_10_ scale. We created “pregnancy” values for DE, DM, and total DAP concentrations by averaging the two log-transformed pregnancy measures. The two total DAP measurements during pregnancy were correlated (*r* = 0.15, *p* = 0.007) and did not differ significantly (paired *t*-test = −0.38, *p* = 0.71). For 22 women, only a single DAP measurement was available.

Statistical methods and base models were similar to those described previously (Eskenazi et al. 2007). To examine the relationship between PON1 and neurobehavioral development at 2 years of age, we constructed separate multiple regression models for *PON1* genotype (*PON1_−108_* and *PON1_192_*) and enzyme measurements (ARYase and POase) and each outcome, adjusting for DAP metabolite concentrations and the covariates included in our previous analysis (Eskenazi et al. 2007). Specifically, all models were adjusted for log_10_-transformed total DAP, DE, or DM concentrations and exact age at assessment, sex, parity, breast-feeding duration (months), HOME score (continuous), maternal PPVT (continuous), and household income in relation to the federal poverty threshold (U.S. Census Bureau 2000). Models with enzyme measurements were also adjusted for temperature during the assay. Multiple linear regression models for Bayley MDI and PDI scores also included psychometrician (*n* = 4) and testing location (office or RV), whereas multiple logistic regression models for clinical-range PDD also included maternal depression (assessed at 12 months postpartum). Covariates in final models were categorized as noted in Table 1 unless otherwise specified above. To preserve the size of the analytic population, each missing covariate value was imputed by randomly selecting a value from participants with nonmissing values. In models of genotype, tests for trend were performed by substituting 0, 1, or 2 to represent the number of *PON1_−108_* T or *PON1_192_* Q alleles. Studies suggest that PON1 status, which takes into account both enzyme level and *PON1_192_* genotype, is useful for investigating the relationship between PON1 and health outcomes in epidemiologic studies (Li et al. 2003; Richter et al. 2010). Therefore, to incorporate a measure of PON1 status, we included variables for both ARYase (as a measure of PON1 enzyme levels) and *PON1_192_* genotype (as a proxy measure of PON1 enzyme activity) within the same statistical models and also considered their interaction.

Potential interactions between maternal urinary DAP concentrations (log-transformed continuous variables) and *PON1* genotypes were explored by adding interaction terms. We also examined interaction by number of variant alleles (0, 1, 2). Models for DAP concentrations and neurobehavioral outcomes stratified by *PON1* genotype are presented. Similarly, interactions between DAP concentrations and PON1 enzyme levels and activity were examined using interaction terms for DAP concentrations and continuous PON1 enzyme measurements; models stratified by tertile of enzyme quantity or activity are presented.

Statistical significance for main effects terms was based on a *p*-value of 0.05 and for interaction terms, on a *p*-value of 0.15. All analyses were performed in STATA 10.0 (StataCorp LP, College Station, TX).

## Results

Most study mothers were young (mean ± SD = 26.2 ± 5.2 years of age at delivery), born in Mexico, and married or living as married (Table 1). Nearly 60% were living at or below the federal poverty threshold. About 44% of the women worked in agriculture during pregnancy, 82% had at least one household member working in agriculture, and 23% lived near a field during pregnancy. Nearly all women breast-fed; median duration was 6 months. The women’s PPVT scores were below the expected standardized average of 100 (mean ± SD = 86 ± 21).

The geometric mean (GM) of the average total DAP levels for the women during pregnancy was 110 nmol/L [95% confidence interval (CI), 101–120] with a larger proportion composed of DM DAP metabolites (GM = 77; 95% CI, 70–85) than of DE DAP metabolites (GM = 18; 95% CI, 16–19). The children’s mean ± SD scores were 85 ± 12 on the Bayley MDI and 98 ± 11 on the Bayley PDI. Based on maternal report on the CBCL, 51 (14%) exceeded the clinical cutoff criteria for PDD (χ^2^ test, *p* < 0.001 comparing 14% observed with 3% expected).

As we previously reported, about half of the children were heterozygous for *PON1_192_* and *PON1_−108_* (Huen et al. 2009a, 2009b), with allelic frequencies for both of approximately 0.5 [see Supplemental Material, Table 1 (doi:10.1289/ehp.1002234)]. *PON1_−108TT_* and *PON1_192QQ_* genotype were associated with mostly lower enzyme measures, suggesting that individuals with these genotypes might be more susceptible to effects of OPs. Neonates with *PON1_−108TT_* genotype (20.3%) had the lowest measures of ARYase and POase among *PON1_−108_* genotypes. Neonates with *PON1_192QQ_* (22.9%) showed lower measures of POase than did neonates with other *PON1_192_* genotypes but no significant difference in ARYase levels (Holland et al. 2006; Huen et al. 2009a). At 2 years of age, average POase activities and ARYase levels were two to three times higher than in cord blood. Two-year-olds showed similar POase enzyme patterns in relation to their genotype as when they were neonates. ARYase levels were somewhat higher in 2-year-olds with *PON1_192QQ_* than in those with other *PON1_192_* genotypes. We observed similar associations between mother’s *PON1* genotype and enzyme measurements [Supplemental Material, Table 2 (doi:10.1289/ehp.1002234)].

**Figure 1:**
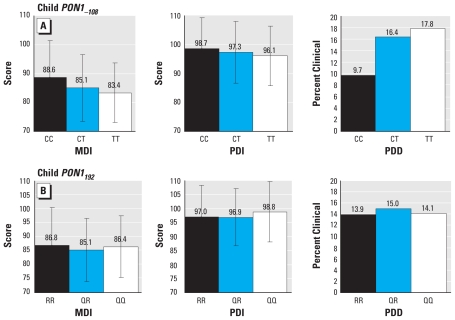
Unadjusted relationship (mean ± SD) of child *PON1_−108_* *(A*) and *PON1_192_* *(B*) genotypes and MDI and PDI scores and maternal report of PDD on the CBCL.

Children with the *PON1_−108T_* allele had lower average MDI and PDI scores and were more likely to be reported by their mothers as having symptoms characteristic of PDD (Figure 1). We saw no clear pattern of associations with outcomes with *PON1_192_* genotype. The *PON1_−108T_* allele remained associated with poorer performance on the Bayley MDI and PDI (Table 2) after adjustment for covariates. Specifically, compared with children with *PON1_−108CC_* genotype, children with *PON1_−108CT_* and *PON1_−108TT_* genotypes performed, respectively, 3.9 points (*p* = 0.004) and 5.7 points (*p* = 0.001) lower on the Bayley MDI (*p*-value for trend = 0.01) and 1.4 (*p* = 0.27) and 2.8 points (*p* = 0.07) lower on the Bayley PDI (*p*-value for trend = 0.07). We noted similar patterns, albeit muted, for maternal genotype for *PON1_−108_* and MDI scores [see Supplemental Material, Table 3 (doi:10.1289/ehp.1002234)]. Children with the *PON1_−108T_* allele were slightly but not significantly more likely to be reported by their mothers as having symptoms consistent with PDD, after controlling for potential confounders. We found no clear relationship between the child’s or mother’s *PON1_192_* genotype and Bayley scores or maternal report of PDD symptoms, except for somewhat better PDI scores among children with *PON1_192QQ_* (*p*-value for trend = 0.10).

Continuous measures of ARYase and POase in maternal, cord, or child blood were not related to neurobehavioral performance at 2 years of age on the Bayley MDI or PDI (Table 3). Cord PON1 measures also were not associated with maternal report of PDD, although 2-year-old ARYase levels [odds ratio (OR) = 0.6; 95% CI, 0.4–0.9] and maternal POase activity (OR = 0.6; 95% CI, 0.4–1.0) were associated with decreased odds of PDD at 2 years of age. Results were similar when we categorized ARYase and POase into tertiles, although those in the highest tertiles of ARYase had the highest MDI scores (*p*_trend_ = 0.04; data not shown). Associations between the outcomes and ARYase were comparable when we included *PON1_192_* genotype in the model, and we found no evidence of statistical interaction (although children with *PON1_192QQ_* and in the highest tertile of ARYase did have the highest adjusted MDI and PDI scores).

Table 4 shows the relation of maternal DAP levels and neurobehavioral outcomes stratified by child genotype. As we previously reported (Eskenazi et al. 2007), maternal DAP concentrations were negatively associated with child MDI scores at 2 years of age, and this relationship was primarily with DM DAPs. The interaction between genotype and DAP concentrations was not statistically significant (interaction *p* = 0.98); however, the inverse association between DAP concentrations and MDI scores was progressively stronger among children with *PON1_−108CT_* and *PON1_−108TT_* genotypes relative to *PON1_−108CC_*. This relationship was most apparent for DM DAP concentrations: For each 10-fold increase in maternal DM DAP levels, we observed a drop in child MDI scores of −2.2 points (*p* = 0.45) for children with *PON1_−108CC_*, −3.4 points (*p* = 0.09) for children with *PON1_−108CT_*, and −5.9 points (*p* = 0.03) for children with *PON1_−108TT_* (interaction *p* = 0.91). We observed a similar trend (albeit nonsignificant) for DE DAP concentrations and MDI scores by genotype. The relation between DAP concentrations and MDI scores did not differ progressively by *PON1_192_* genotype.

We previously found no relation between maternal DAP concentrations and child PDI scores at 2 years of age, and we also observed no clear relation when we stratified by child *PON1_−108_* (Table 4). However, we found an inverse association between DAP concentrations and PDI scores among 2-year-olds with *PON1_192QQ_* (*p* = 0.10) and an interaction of DE DAP concentrations and PDI scores by *PON1_192_* genotype (interaction *p* = 0.14).

Although we observed a strong relation of maternal DAP concentrations and maternal report of PDD overall, we found no clear difference in the odds across strata of *PON1_−108_* and only a suggestion of a higher odds in children with *PON1_192QQ_* or *PON1_192RR_* (for DMs). However, numbers within each stratum are too small to provide reliable ORs, and none of the interactions between DAP concentrations and genotype was statistically significant. We observed similar, albeit weaker, associations between DAP concentrations and neurobehavioral outcomes when we stratified results by maternal genotype rather than child genotype [Supplemental Material, Table 4 (doi:10.1289/ehp.1002234)].

We did not observe statistically significant interaction between DAPs and enzyme measurements (as a continuous measure) in relation to any of the neurobehavioral end points. We present the relation of maternal DAP concentrations and neurobehavioral outcomes within tertiles of cord and maternal ARYase and POase in Supplemental Material, Tables 5 and 6 (doi:10.1289/ehp.1002234). (We do not present the results stratified by 2-year-old PON1 measurements because the biological mechanism of *in utero* exposure being modified by child’s enzyme levels 2 years later would be uncertain.) Consistent with results by child *PON1* genotype, the relation of DAP concentrations and child MDI scores was strongest within the group of children with the lowest tertiles of cord ARYase and POase, although the pattern was not as clear. We observed slightly stronger relationships of PDI scores and maternal DAP concentrations, particularly for DE DAPs, among children with cord blood in the lowest tertile of ARYase relative to children with higher cord blood PON1 activity. Maternal DAP concentrations were consistently positively associated, although not significantly, with reports of PDD across all tertiles of cord ARYase activity. However, the relationship of DAP concentrations and PDD was significant only in the group with the lowest tertile of POase. Again, relatively small numbers in each tertile made these estimates unreliable. Results for DAP concentrations and MDI scores, PDI scores, and PDD assessment across maternal enzyme levels and activities [Supplemental Material, Table 5 (doi:10.1289/ehp.1002234)] were similar to those for cord blood enzyme levels.

## Discussion

Our previous study demonstrated that women’s levels of OP pesticide DAP metabolites in urine during pregnancy are related to their 2-year-olds’ mental development and reported symptoms of PDD (Eskenazi et al. 2007). In the present study, we examined whether these associations were modified by the mother and child’s PON1 genotypes and associated enzyme levels and activities. In analyses of PON1 and neurobehavior, we found that the *PON1_−108T_* allele in children was associated with poorer Bayley MDI scores and with somewhat poorer Bayley PDI scores. Maternal, cord, and child PON1 enzyme levels and activities, although related to genotype, were not significantly associated with these neurobehavioral outcomes except that children were less likely to display symptoms of PDD when they or their mothers had higher ARYase levels or POase activities.

Although we have observed a relationship of DAP concentrations and neurobehavioral outcomes and of PON1 and these same end points, we are less certain as to whether genotype or enzyme measurements modify the association of DAP concentrations and neurobehavior—that is, whether a subgroup of children by virtue of their genetic makeup are more susceptible to the adverse effects of maternal exposure to OP pesticides during pregnancy. There is a suggestion with MDI scores that children with *PON1_−108T_* allele show a stronger association with OP pesticide exposure *in utero* (as measured by maternal DAPs), but the interaction is not significant.

PON1 has been implicated in the etiology of a number of adult-onset neurologic diseases such as Parkinson and Alzheimer diseases (Erlich et al. 2006; Leduc and Poirier 2008; Paragh et al. 2002; Zintzaras and Hadjigeorgiou 2004). In addition, PON1 polymorphisms and enzyme activities have been associated with autism spectrum disorder. In a family-based linkage study, D’Amelio et al. (2005) found that Caucasian-American, but not Italian, patients diagnosed with autism were more likely to carry the *PON1_192R_* allele and more likely, although not significantly, to carry the *PON1_−108T_* allele. In line with these results, Pasca et al. (2010) reported that ARYase levels and POase activity were significantly lower in 50 Romanian autistic children compared with 30 age- and sex-matched nonautistic controls, but they observed no differences in *PON1_192_* or *PON1_55_* allelic frequencies between groups (and they did not examine *PON1_−108_*). Another study (Serajee et al. 2004) also failed to find a relation of *PON1_55_* allelic frequency in a family-based association study of 196 trios but did not examine *PON1_−108_* or *PON1_192_*.

In the above studies, autistic patients were diagnosed by clinicians. In the present study, we based PDD on maternal report of symptomatology associated with autism-like behavior, and these reports were not confirmed by a clinician’s assessment. Nevertheless, for the most part, our findings are in line with those reported by Pasca et al. and D’Amelio et al. (2005): We observed a nonsignificant increase in maternal report of PDD in primarily Mexican-American children with the *PON1_−108T_* allele but not with the *PON1_192R_* allele, and we found that 2-year-olds with higher ARYase levels and children whose mothers had higher ARYase or POase measures during pregnancy were less likely to be reported by their mother as having PDD.

D’Amelio et al. (2005) hypothesized that genetic vulnerability in the presence of exposure to OP pesticides may contribute to the development of childhood autism, especially in North America, where until recently OP pesticides have been more commonly used in homes than in Europe. OP pesticides can induce oxidative stress (Milatovic et al. 2006), and PON1 may protect the body from the effects of OP pesticides both through its antioxidant properties and its ability to metabolize the activated form (e.g., chlorpyrifos-oxon) of the pesticides. We previously reported that with every 10-fold increase in DAP metabolites concentrations in the urine of the women in this study during pregnancy, we found more than a doubling in odds of their reporting signs of PDD in their 2-year-olds. Nevertheless, we observed no evidence that PON1 genotype or enzyme activity modifies this association as suggested by the hypothesis of D’Amelio et al. (2005). However, given the lack of clinical diagnosis and that our sample sizes when stratified by *PON1* genotype were relatively small, this hypothesis cannot be rejected.

Few studies have tested the hypothesis that *PON1* genotype modifies the effects of pesticide exposure on neuropsychologic functioning. One study from Israel demonstrated increased frontal cortical brain activity and decreased temporal lobe activity in pesticide-exposed workers with *PON1_192R_* compared with either those without the R allele or unexposed adults (Browne et al. 2006). Only one previous study has examined the ability of PON1 enzyme to modify the association of OP pesticide exposure and neurobehavioral end points in children. Engel et al. (2007) found that DAP metabolite levels during pregnancy were associated with an increase in abnormal reflexes in the neonates as assessed on the Brazelton Neonatal Assessment Scale, and the association between DAP concentrations, specifically DM DAPs, and abnormal reflexes was strongest in the infants born to women with the lowest levels of ARYase.

In the present study, we extend the observations of Engel et al. (2007) to toddlers. Although we find a clear relationship of *PON1_−108_* genotype with MDI scores and, to a lesser extent, PDI scores and a trend of increasingly stronger associations of maternal DAPs and MDI scores, we cannot provide firm evidence that the *PON1* genotype or phenotype modifies the relation of exposure and mental development. Recent studies demonstrate that PON1 is a multifunctional enzyme, and it plays a significant role in protection against oxidative stress and lipid peroxidation in addition to OP detoxification. Thus, it is possible that the relationship between PON1 and mental development is more strongly influenced by the role of PON1 in protection from oxidative stress than by its OP pesticide detoxification capabilities, which may explain the absence of strong effect modification. Further, other pesticides not hydrolyzed by PON1, including malathion (devolves to DM DAPs), have been shown to induce oxidative stress (Durak et al. 2009; Franco et al. 2009). This may explain why we observed stronger associations of neurobehavior with DM DAPs than with DE DAPs (after stratification by *PON1* genotype) despite the fact that DEs are derived from known PON1 substrates (chlorpyrifos-oxon and diazoxon).

Our research is limited in a number of ways. DAP metabolites are an imperfect measure of OP pesticide exposure given the short half-life of OP pesticides, the variability in exposure over time, differences in metabolism that may affect excretion (Wessels et al. 2003), and the potential for exposure to preformed DAPs in the environment (Quirós-Alcalá 2010). In fact, it is possible that because the *PON1* gene is involved with the ability to metabolize OP pesticides, levels of excreted DAPs may be related both to exposure and to genetics. We are currently examining this issue by analyzing the interrelationship of blood OP pesticide measurements, genotype, and urinary DAP concentrations in this cohort. Our study results may not be generalizable given this unique cohort of primarily Mexican-American children from an agricultural area. Other populations may have a different spectrum of OP pesticide exposures and *PON1* allelic distributions, which are known to vary widely among different ethnic groups (Chen et al. 2003; Rojas-Garcia et al. 2005). The relationship between genotype and enzyme activities in adults, however, appears to be comparable in different ethnic groups (Costa et al. 2005).

Another limitation is that we have constructed a fairly simple model for neurobehavioral development. Although we have examined and/or controlled for a number of key potential confounders, neurobehavioral development is complex and multifactorial, and numerous genes and environmental agents, social, biological, physical, and chemical, are likely involved. In addition, when data are stratified by genotype, the fairly large numbers of this study become relatively small in each stratum, making it difficult to find significant statistical interactions.

We have not examined other genes that have been implicated in the hypothesized gene–environment interaction such as *RELN* or *AChE* or other *PON1* polymorphisms (Moy and Nadler 2008). We have recently shown that other *PON1* polymorphisms are also associated with ARYase and POase measurements in mothers and children of the CHAMACOS cohort; however, most of them were in strong linkage disequilibrium with those examined in this report (Huen et al. 2010) and are unlikely to affect the associations noted here. Haplotype analysis is sometimes proposed as a more informative approach for analysis of genetic effects, but we did not see an improvement of association with neurodevelopment using haplotypes in this study (data not shown). Additionally, we did not find a significant difference in functional effects of *PON1* haplotypes compared with multiple polymorphisms in predicting PON1 enzyme measurements (Holland et al. 2006; Huen et al. 2010).

The strengths of this study are that we have focused on an agricultural population with relatively high levels of OP pesticide exposures, that we have measured this exposure during pregnancy in this longitudinal birth cohort study, and that we have genotyped and measured enzyme activity in mothers and children at birth and during infancy.

In summary, we have found that *PON1_−108T_* allele is related to mental development and, to a lesser extent, psychomotor development in toddlers. This study adds to the growing evidence that the *PON1* gene is associated with an array of neurologic end points in adults and in children. Although we did not observe a statistical interaction between prenatal OP pesticide exposure as measured by pesticide metabolites and PON1, we did observe a consistent trend, and our results need replication in other populations and perhaps pooling with similar studies to provide adequate sample sizes.

## Figures and Tables

**Table 1:** Demographic characteristics of CHAMACOS mothers with children followed to 2 years of age: Salinas Valley, California, 2000–2001 (*n* = 371).

Characteristic	*n* (%)
Child sex	
Female	189 (50.9)
Male	182 (49.1)
Maternal age at delivery (years)	
18–24	153 (41.2)
25–29	123 (33.2)
30–34	63 (17.0)
≥ 35	32 (8.6)
Marital status during pregnancy	
Married/living as married	307 (82.8)
Single	64 (17.3)
Parity	
0	117 (31.5)
≥ 1	254 (68.5)
Maternal education during pregnancy	
< 6th grade	163 (43.9)
7th–12th grade	135 (36.4)
Completed high school	73 (19.7)
Maternal country of birth	
Mexico	323 (87.1)
USA	44 (11.9)
Other	4 (1.1)
Alcohol use during pregnancy	
Yes	4 (1.1)
No	354 (98.9)
Smoking during pregnancy	
Smoker	17 (4.6)
Nonsmoker living with smoker	28 (7.6)
Nonsmoker	326 (87.9)
Maternal depressive symptoms (CES-D ≥ 16) at 1 year	
Yes	181 (50.8)
No	175 (49.2)
Breast-feeding at 2 years	
Yes	30 (8.1)
No	341 (91.9)
Household income^a^	
≤ Federal poverty threshold	218 (58.8)
> Federal poverty threshold	153 (41.2)
Worked in agriculture during pregnancy	
Yes	163 (44.2)
No	206 (55.8)
Lived with agricultural worker(s) during pregnancy	
Yes	302 (82.1)
No	66 (17.9)
Lived within 200 feet of agricultural field	
Yes	85 (23.0)
No	284 (77.0)

Percentages represent those of known values. Values are limited to participants who had a Bayley assessment or maternal CBCL report performed at 24 months.

a Poverty level compares federal poverty thresholds with household income divided by the number of people supported (U.S. Census Bureau 2000).

**Table 2:** Adjusted^a^ association of child *PON1_−108_* and *PON1_19_*_2_ genotypes and neurobehavioral development at 2 years of age: CHAMACOS study, Salinas Valley, California, 2000–2003.

Genotype	*n*^b^	Bayley MDI β (95% CI)	*p*-Value trend	Bayley PDI β (95% CI)	*p*-Value trend	CBCL PDD OR (95% CI)	*p*-Value trend
*PON1_−108_*							
CC	111	Reference	< 0.01	Reference	0.07	Reference	0.14
CT	179	−3.9 (−6.6 to −1.2)#		−1.4 (−3.8 to 1.0)		1.5 (0.7 to 3.3)	
TT	74	−5.7 (−9.0 to −2.5)#		−2.8 (−5.7 to 0.2)*		2.0 (0.8 to 5.1)	
*PON1_192_*							
RR	94	Reference	0.65	Reference	0.10	Reference	0.94
QR	188	−0.5 (−3.4 to 2.4)		0.3 (−2.2 to 2.9)	1.0	(0.4 to 2.2)	
QQ	86	0.7 (−2.6 to 4.0)		2.4 (−0.5 to 5.4)	1.0	(0.4 to 2.4)	

a Adjusted for prenatal DAPs and age at assessment, sex, parity, breast-feeding duration, HOME score, maternal PPVT, and household poverty status. Bayley MDI and PDI models also included psychometrician and testing location; PDD models also included maternal depression.

b Numbers may vary slightly depending on the model.

* *p* < 0.10.

# *p* < 0.01.

**Table 3:** Adjusted^a^ association of PON1 enzyme measurements (per SD increase) in cord and child blood and neurobehavioral development at 2 years of age: CHAMACOS study, Salinas Valley, California, 2000–2003.

Enzyme	Mean ± SD	Bayley MDI [β (95% CI)]	Bayley PDI [β (95% CI)]	CBCL PDD [OR (95% CI)]
Fetal enzymes (*n* = 265)				

ARYase	34.0 ± 16.8	0.8 (−0.6 to 2.1)	−0.6 (−1.8 to 0.7)	0.8 (0.6 to 1.2)
POase	254.3 ± 165.0	0.1 (−1.3 to 1.5)	−1.0 (−2.2 to 0.2)	1.0 (0.7 to 1.4)

2-year-old enzymes (*n* = 249)				

ARYase	87.0 ± 25.1	0.4 (−1.0 to 1.8)	0.1 (−1.2 to 1.3)	0.6 (0.4 to 0.9)**
POase	665.2 ± 385.8	−0.2 (−1.6 to 1.3)	−0.5 (−1.7 to 0.8)	0.8 (0.6 to 1.3)

Maternal enzymes during pregnancy (*n* = 302)				

ARYase	133.3 ± 40.9	0.7 (−0.7 to 2.1)	0.4 (−0.8 to 1.7)	0.7 (0.5 to 1.0)*
POase	958.6 ± 599.3	−0.1 (−1.4 to 1.2)	0.0 (−1.2 to 1.2)	0.6 (0.4 to 1.0)**

a Adjusted for prenatal DAPs and age at assessment, sex, parity, breast-feeding duration, HOME score, maternal PPVT, household poverty status, and assay temperature. Bayley MDI and PDI models also included psychometrician and testing location; PDD models also included maternal depression.

* *p* < 0.10.

** *p* < 0.05.

**Table 4:** Adjusted^a^ associations of prenatal DAPs and neurobehavioral development at 2 years of age, stratified by child *PON1_−108_* and *PON1_19_*_2_ genotype: CHAMACOS study, Salinas Valley, California, 2000–2003.

DAP	Bayley MDI β (95% CI)	*p*-Value interaction^b^	Bayley PDI β (95% CI)	*p*-Value interaction^b^	CBCL PDD OR (95% CI)	*p*-Value interaction^b^
Total DAPs						

*PON1_−108_*						
CC	−3.2 (−9.8 to 3.5)	0.98	−2.3 (−7.8 to 3.3)	0.89	4.2 (0.5−36.8)	0.91
CT	−3.7 (−8.0 to 0.6)*		−0.8 (−4.8 to 3.3)		2.0 (0.6–6.0)	
TT	−5.5 (−11.1 to 0.1)*		−1.0 (−7.1 to 5.1)		1.9 (0.3–10.4)	
*PON1_192_*						
RR	−6.5 (−15.6 to 2.6)	0.33	−1.7 (−8.7 to 5.4)	0.53	5.4 (0.7–44.0)	0.29
QR	−1.2 (−5.2 to 2.9)		0.1 (−3.5 to 3.8)		1.2 (0.4–3.6)	
QQ	−6.9 (−12.8 to −0.9)**		−5.1 (−11.1 to 1.0)*		5.2 (0.8–35.1)*	

DM DAPs						

*PON1_−108_*						
CC	−2.2 (−8.0 to 3.6)	0.91	−1.6 (−6.4 to 3.3)	0.87	3.3 (0.5–21.3)	0.94
CT	−3.4 (−7.4 to 0.6)*		−0.3 (−4.0 to 3.4)		2.2 (0.8–5.9)	
TT	−5.9 (−11.1 to −0.6)**		−1.2 (−6.9 to 4.4)		1.9 (0.4–9.8)	
*PON1_192_*						
RR	−4.4 (−12.4 to 3.6)	0.38	−2.1 (−8.3 to 4.0)	0.36	4.8 (0.8–31.1)*	0.20
QR	−1.3 (−4.9 to 2.4)		0.7 (−2.6 to 4.0)		1.2 (0.5–3.3)	
QQ	−7.4 (−13.0 to −1.9)**		−4.7 (−10.4 to 1.0)		6.1 (1.0–39.3)*	

DE DAPs						

*PON1_−108_*						
CC	−0.3 (−7.2 to 6.7)	0.84	0.9 (−4.9 to 6.8)	0.66	7.4 (0.6–93.9)	0.44
CT	−1.7 (−6.3 to 3.0)		−2.2 (−6.5 to 2.1)		0.8 (0.2–2.8)	
TT	−3.4 (−8.8 to 2.1)		−1.5 (−7.3 to 4.2)		0.8 (0.1–4.3)	
*PON1_192_*						
RR	1.4 (−8.4 to 11.1)	0.47	4.5 (−2.9 to 11.9)	0.14	1.0 (0.1–8.2)	0.97
QR	−1.1 (−5.2 to 3.0)		−1.9 (−5.6 to 1.8)		0.8 (0.2–2.6)	
QQ	−2.5 (−8.7 to 3.6)		−3.8 (−9.9 to 2.3)		1.2 (0.2–7.7)	

a Adjusted for age at assessment, sex, parity, breast-feeding duration, HOME score, maternal PPVT, and household poverty status. Bayley MDI and PDI models also included psychometrician and testing location; PDD models also included maternal depression.

b Interaction *p*-value was calculated using a postestimation combined *F*-test (or chi-square test) for the two interaction variables between genotype and DAPs (e.g., 192_QR_ × DAP and 192_QQ_ × DAP). Conclusions regarding interaction were similar if a single interaction term with the number of C or Q alleles (0, 1, 2) was used instead.

* *p* < 0.10.

** *p* < 0.05.
